# Complete Genome Sequence of *Streptococcus thermophilus* MN-BM-A02, a Rare Strain with a High Acid-Producing Rate and Low Post-Acidification Ability

**DOI:** 10.1128/genomeA.00979-15

**Published:** 2015-09-03

**Authors:** Yudong Shi, Yun Chen, Zhouyong Li, Lan Yang, Wei Chen, Zhishen Mu

**Affiliations:** Research and Development Center, Inner Mongolia Mengniu Dairy (Group) Co. Ltd., Hohhot, Inner Mongolia, China

## Abstract

*Streptococcus thermophilus* MN-BM-A02 was originally isolated from a traditional fermented dairy product in China. The characteristics of this bacterium are its high acid-producing rate and low post-acidification. This study presents the genome sequence of MN-BM-A02. Its complete genome comprises 2,025 genes and 1,850,434 nucleotides with an average G+C content of 39%.

## GENOME ANNOUNCEMENT

*Streptococcus thermophilus* is a lactic acid bacterium widely used by the dairy industry as a starter culture to obtain high-quality fermented products, such as yogurt and cheeses (1). In the process of processing, transportation, and storage of fermented products such as yogurt, *Streptococcus thermophilus* can metabolize lactose to produce lactic acid, affecting the quality of yogurt, the time of milk coagulation, and shelf life (2).

*S. thermophilus* MN-BM-A02 was originally isolated from a traditional fermented dairy food called Dairy Fan, originating from the Dali region of the Yunnan province, China. The high acid-producing rate and low post-acidification ability are good fermentation characteristics of MN-BM-A02, which could shorten milk coagulation time and extend the shelf life of fermented dairy products.

The whole genome of *S. thermophilus* strain MN-BM-A02 was sequenced with a combined strategy of 454 sequencing (3) and Solexa paired-end sequencing technology (4). Genomic libraries containing 9-kb inserts were constructed, and 132,195 paired-end reads and 230,299 single-end reads were generated using the GS FLX system, giving 71-fold coverage of the genome. The majority (97.2%) of reads were assembled into one large scaffold, including 42 nonredundant contigs, using the 454 Newbler assembler (454 Life Sciences, Branford, CT). A total of 6,282,548 reads (500-bp library) were generated to reach a depth of 340-fold coverage with an Illumina Solexa GA IIx (Illumina, SanDiego, CA) and mapped to the scaffolds using Burrows-Wheeler alignment (BWA) (5). The gaps between scaffolds were filled by sequencing PCR products using an ABI 3730 capillary sequencer. The genome analysis was performed as described previously (6, 7).

The complete genome sequence of MN-BM-A02 contains a circular 1,850,434-bp chromosome with a G+C content of 39%. There are 2,025 genes in total, including 1,904 coding genes, 49 pseudogenes, 5 rRNA operons, and 57 tRNAs in the MN-BM-A02 genome.

### Nucleotide sequence accession number.

The complete annotated genome sequence of *Streptococcus thermophilus* MN-BM-A02 was deposited in GenBank under the accession no. CP010999.
